# Assessment of the psychometric properties and refinement of the Health and Self-Management in Diabetes Questionnaire (HASMID)

**DOI:** 10.1186/s12955-020-01305-3

**Published:** 2020-03-05

**Authors:** Jill Carlton, Donna Rowen, Jackie Elliott

**Affiliations:** 1 School of Health and Related Research (ScHARR), University of Sheffield, Regent Court, 30 Regent Street, Sheffield, S1 4DA UK; 2 Department of Oncology and Metabolism, University of Sheffield, Medical School, Sheffield, S10 2JF UK; 3 Sheffield Teaching Hospitals NHS Trust, Diabetes and Endocrine Centre, Northern General Hospital, Sheffield, S5 7AU UK

**Keywords:** Diabetes mellitus, Self-management, Patient reported outcome (PRO), Psychometric, Quality of life (QoL), Questionnaire

## Abstract

**Background:**

The Health And Self-Management In Diabetes (HASMID^v1^) questionnaire consists of 8 attributes, 4 about quality of life, and 4 about self-management. The overall aim of this study was to rigorously examine the psychometric properties of the HASMID^v1^ questionnaire.

**Methods:**

The study comprised two phases. Phase 1 identified items of the HASMID^v1^ questionnaire that potentially required rewording through consultation with a patient involvement panel and two focus groups of people with diabetes. Phase 2 involved a cross-sectional longitudinal survey where HASMID, EQ-5D-5L, health, treatment and sociodemographic questions were administered using both paper and online versions to people with diabetes. Participants were asked to complete the survey again approximately 3 months later. Psychometric analyses were undertaken to examine floor and ceiling effects, item distributions, known group differences and internal consistency. Rasch analysis was undertaken to assess differential item functioning and disordered thresholds.

**Results:**

Phase 1 derived five alternative wordings to items: Irritable, Affects Mealtimes, Daily Routine, Social Activities and Problem. Phase 2 achieved 2835 responses at time point 1 (*n* = 1944 online, *n* = 891 paper version) and 1243 at time point 2 (*n* = 533 online, *n* = 710 paper version). Overall the HASMID items performed well, though two alternative worded items (Irritable and Social Activities) provided additional information not fully captured by the original HASMID items.

**Conclusion:**

Psychometric evaluation and Rasch analysis were used in conjunction with expert opinion to determine the final questionnaire. The application of psychometric analyses or Rasch analysis alone to inform item selection would have resulted in different items being selected for the final instrument. The benefit of a combined approach has produced an instrument which has a broader evaluation of self-management. The final validated HASMID-10 is a short self-report PRO that can be used to evaluate the impact of self-management for people living with diabetes. HASMID-10 can be scored using total summative scores, with utility and monetary values also available for use in cost-utility and cost-benefit analyses.

## Background

Diabetes is a complex condition in which the long-term consequences of the disease are largely determined by the ability of the individual to self-manage dietary habits, physical activity and various medications. In terms of glucose control, people with diabetes (PwD) need to avoid chronic hyperglycaemia (high glucose levels in the bloodstream) to evade microvascular diabetes-related complications which include eye damage (retinopathy), kidney damage (nephropathy) and neuropathy (nerve damage), which if severe can lead to blindness, dialysis and leg amputations, respectively. Macrovascular complications such as heart attacks and strokes are also increased as a result of chronic hyperglycaemia. Some medications can cause hypoglycaemia (low blood glucose levels in the bloodstream), which if mild may cause for example sweating, slurred speech and tingling, but if more severe can cause confusion, loss of consciousness, seizures and occasionally may be fatal. Many people with type 2 diabetes (T2DM) control their glucose through adapting their diet or taking tablets that do not cause hypoglycaemia. For some, with longer duration of T2DM, medications may be required which if not given at just the right dose may cause hypoglycaemia (including insulin). For people with type 1 diabetes (T1DM), insulin treatment is the only option, and doses need to be adjusted according to the amount of carbohydrate in each meal/drink (as this is the primary source of glucose), the current glucose level, the intensity of physical activity (prior and post the injection) and whether or not there has been any recent alcohol consumption. As there are several factors to take into consideration multiple times each day it is not unsurprising that avoiding hyper- and hypo-glycaemia is more challenging in T1DM than in T2DM. In all forms of the disease (T1DM accounts for approximately 5%, T2DM approximately 90%, other approximately 5%), additional aspects of diabetes care may include medication for raised blood pressure and/or raised cholesterol, as well as therapy for the diabetes-related complications. Hence, the ability of the patient to self-manage their condition is of paramount importance to help them live as healthy a life as possible. Some PwD live a normal life-span with minimal complications of diabetes, whereas others suffer devastating complications and die early [1]. Therefore, educational interventions which aim to improve self-management skills such as DESMOND for T2DM, and DAFNE [2] for T1DM, are seen as the cornerstone of good diabetes care [3].

In a previous study, we confirmed that self-management plays a significant role in the life of PwD [4]. During the project we developed a patient reported outcome (PRO) measure that was able to measure the quality of life impact of self-managing the condition capturing both health and treatment experience. The Health And Self-Management In Diabetes (HASMID^v1^) questionnaire consists of 8 attributes, 4 about quality of life, and 4 about self-management [4]. However, psychometric analyses around the performance of the questionnaire have not been previously undertaken. The validation of all PROs is important to demonstrate that they measure what they are intended to measure. The psychometric performance of PROs should be assessed and reported so that users can be assured that the instrument is appropriate to use. The overall aim of this study was to rigorously examine the psychometric properties of the HASMID^v1^ questionnaire. The study had two phases: Phase 1 identified any items of the HASMID^v1^ questionnaire that may require rewording and deriving alternative and any additional items; Phase 2 conducted an assessment of the measurement properties of the HASMID^v1^ questionnaire in a large observational survey of people with diabetes. The aim was to examine floor/ceiling effects, known-group differences and internal consistency.

## Methods

### HASMID^v1^ questionnaire

The questionnaire consists of 8 items with four response options (never, sometimes, usually and always). Response options are scored from 1 to 4 with a higher score indicating more severe impact upon quality of life. The scoring of the questionnaire is detailed in Table 1. The overall questionnaire is reverse scored from 0 to 24, with a high score indicating better health related quality of life, and a lower score indicating worse health related quality of life [4]. The measure has been valued using discrete choice experiments that enable the measure to be used to generate quality adjusted life years (QALYs) for use in cost-utility analyses, or willingness-to-pay values to generate monetary values for use in cost-benefit analyses [5, 6].

**Table 1:** HASMID^v1^ questionnaire

Dimension	Score	Wording
Mood	3	You never find yourself losing your temper over small things
	2	You sometimes find yourself losing your temper over small things
	1	You usually find yourself losing your temper over small things
	0	You always find yourself losing your temper over small things
Hypoglycaemic attacks	3	You never worry about going hypo
	2	You sometimes worry about going hypo
	1	You usually worry about going hypo
	0	You always worry about going hypo
Vitality	3	You are never tired
	2	You are sometimes tired
	1	You are usually tired
	0	You are always tired
Social Limitations	3	Your days are never tied to meal times
	2	Your days are sometimes tied to meal times
	1	Your days are usually tied to meal times
	0	Your days are always tied to meal times
Control	3	You feel you have a lot of control of your diabetes
	2	You feel you have some control of your diabetes
	1	You feel you have little control of your diabetes
	0	You feel you have no control of your diabetes
Hassle	3	You find your life with diabetes is never a hassle
	2	You find your life with diabetes is sometimes a hassle
	1	You find your life with diabetes is often a hassle
	0	You find your life with diabetes is always a hassle
Stress	3	You find your life with diabetes is never stressful
	2	You find your life with diabetes is sometimes stressful
	1	You find your life with diabetes is often stressful
	0	You find your life with diabetes is always stressful
Support (All support you have; from family, friends and health care professionals)	3	You feel totally supported with your diabetes
	2	You feel you have a lot of support with your diabetes
	1	You feel you have a little support with your diabetes
	0	You feel you have no support with your diabetes
Additional items generated from Phase 1		
Mood	3	You are never irritable
	2	You are sometimes irritable
	1	You are usually irritable
	0	You are always irritable
Social Limitations	3	Your diabetes never affects your meal times
	2	Your diabetes sometimes affects your meal times
	1	Your diabetes usually affects your meal times
	0	Your diabetes always affects your meal times
Social Limitations	3	Your diabetes never affects your daily routine
	2	Your diabetes sometimes affects your daily routine
	1	Your diabetes usually affects your daily routine
	0	Your diabetes always affects your daily routine
Social Limitations	3	Your diabetes never limits your social activities
	2	Your diabetes sometimes limits your social activities
	1	Your diabetes usually limits your social activities
	0	Your diabetes always limits your social activities
Hassle	3	Your diabetes never causes you a problem
	2	Your diabetes sometimes causes you a problem
	1	Your diabetes usually causes you a problem
	0	Your diabetes always causes you a problem

#### Phase 1

The aim of Phase 1 was to critically evaluate the wording of the HASMID^v1^ questionnaire, and to consider whether any of the existing items could be rephrased. This was undertaken through presentation of the HASMID^v1^ questionnaire to a Patient and Public Involvement (PPI) panel, who advised on aspects of wording on the questionnaire (including wording and meaning of items, response options and instructions), as well as the wording and clarity of participant information sheets and consent forms for the subsequent focus groups. The PPI panel was a pre-existing panel accessed through a local hospital. Members of the panel are volunteers and self-selected. Two focus groups were conducted with PwD. The inclusion criteria for focus group participants were: clinical diagnosis of diabetes; aged 18 years or greater; fluent in English; and able to provide informed consent. Potential participants were identified through an existing University research database (Patients as Educators Programme). The database consists of patients with a clinical diagnosis of diabetes who have volunteered to assist with research projects and staff and medical training. People on the database have consented to be contacted about research projects. Potential participants for the focus groups were identified by administrators of the Patients as Educators Programme, and potential participants were contacted by telephone to see if they wished to participate. If an individual expressed an interest, an information sheet was sent to the potential participant by post. All participants of the focus groups provided written consent prior to the focus groups. Focus groups were held at the local hospital, and facilitated by an experienced qualitative researcher (JC). Each focus group was recorded and transcribed verbatim at a later date.

At the focus groups participants were asked to comment upon a number of aspects of the HASMID^v1^ questionnaire. This included the wording of the instructions for completing the questionnaire; consideration of the wording and meaning of existing items and response options; and suggestions (if any) for refining items. Participants were also asked if there were any other aspects of living with diabetes that were not covered by the HASMID^v1^ questionnaire. Feedback was also requested on the layout of the questionnaire itself, including size and style of font.

A balanced sample was broadly achieved with respect to gender and diabetes type (see Additional Material, Table 1). It should be recognised that no participant was aged less than 50 years. This is possibly due to the timing of the focus group session; both of the focus groups were conducted on a weekday during office hours. It is possible that younger people may have been willing to participate but were unable to do so due to work and/or family commitments. Both focus groups were successful in that each individual actively participated in discussion.

Participants made a number of suggestions regarding the layout and instructions for the HASMID^v1^ questionnaire (summarised in Box 1 in Additional Material). Based on the comments from the focus groups (see Additional Material, Table 2) additional items were formed to test in Phase 2. One alternatively worded item was generated for mood, three for social limitations, and one for hassle (see Table 1). No new items were suggested.

#### Phase 2

The aim of Phase 2 was to evaluate the psychometric properties of the HASMID^v1^ questionnaire, and to determine whether the alternative items identified in Phase 1 performed better than the original HASMID^v1^ items, therefore suggesting whether any modifications were needed.

#### Recruitment

This project sought to formally test the developed HASMID ^v1^ questionnaire (with the additional alternatively phrased items generated in Phase 1). We conducted a longitudinal survey of PwD (including both Type 1 and Type 2). Both online and paper versions of the questionnaire were tested. Potential participants were recruited via four main cohorts:

- DAFNE Online (see http://www.dafneonline.co.uk/), a panel of over 1500 DAFNE graduates; a website designed specifically for people with T1DM who have undertaken a Dose Adjustment for Normal Eating (DAFNE) structured education course, but also accessible to anyone wishing to find out more about T1DM. Recruited via online link providing direct access to the online survey
- Diabetes UK (see http://diabetes.org.uk/); the main charity for all patients with diabetes in the UK. Members received an electronic link in their online newsletter and a printed link on their printed newsletter to the online survey
- A panel of over 2300 patients at Sheffield Teaching Hospitals NHS Trust who consented to be contacted for research studies in diabetes. Potential participants were randomly allocated to participate in either the online or paper-version of the survey. Postal participants were sent information sheets and the questionnaire via post and returned this in a pre-paid envelope. Online participants were sent an Invitation to Participate letter providing information on how to access the online survey.
- Social media through Twitter and a University of Sheffield mailing list, with a link to the online survey.

All consenting participants were given the option to enter a prize draw, with one £50 voucher randomly selected for each 50 participants.

#### Data collection

All participants were asked to complete the HASMID^v1^ questionnaire, EQ-5D-5L, and sociodemographic and health questions (time point 1). Both online and postal respondents were asked if they would be willing to complete the survey again in approximately 3 months’ time (time point 2), for another chance of “winning” a £50 shopping voucher. Those who completed the postal survey were sent an additional pack by Sheffield Teaching Hospitals NHS Trust. Those who completed the online survey were sent a reminder email.

#### Analysis

Standard descriptive analysis was undertaken on the sample, with sub analysis on mode of administration of the survey. The psychometric properties of the HASMID questionnaire (and modified items) were explored. The analyses are detailed below.

### Floor and ceiling effects

Floor and ceiling effects describe the disproportionate numbers of responses given at either end of the scale. A high percentage of floor or ceiling responses may limit the ability to detect change within an instrument.

### Validity

Validity was assessed by examining known-group differences for each item across T1DM and T2DM respondents. The difference in the means between groups, effect size (using Cohen’s d) and Wilcoxon rank-sum (Mann-Whitney) tests were undertaken to assess item performance. The standard effect size value can be described to fall within the ranges of small 0.2 to 0.5, medium 0.5 to 0.8 and large > 0.8. A value of < 0.2 is considered nonsignificant [7]. It was hypothesised that respondents with T1DM would report lower QoL than respondents with T2DM, as previously reported with other measures [8].

### Rasch analysis

Rasch analysis is a logit modelling technique that can be used to inform the selection of items in a patient reported outcome measure [9] and can be used to select best performing items (for example [10–13]). Rasch analysis was used to assess differential item functioning (DIF). DIF is a form of bias across groups of respondents occurring when different groups within the same sample (despite equal severity of the underlying characteristic of health/self-management) respond in a different manner to an individual item. Rasch analysis was applied to identify whether DIF was present by gender, age, and type of diabetes mellitus (DM). DIF by type of DM may not be a reason for removing an item, but is indicative that the item performs differently across T1DM and T2DM. For this study Rasch techniques were applied to a subset of randomly selected participants (*n* = 500) for time point 1.

#### Item selection

The results of the psychometric evaluation and Rasch analysis were used to inform final item selection for the HASMID questionnaire. Modified items were directly compared to the original item, such that: Temper versus Irritable; Hassle versus Problem; and Tied To Mealtimes versus Affects mealtimes, Daily Routine, Social Activities. Item selection was informed by consideration of floor and ceiling effects; DIF; and clinical opinion.

### Internal consistency

The internal consistency of the final questionnaire was assessed by calculating Chronbach’s alpha (α) of the scale at time point 1. Inter-item covariance was calculated in addition to Cronbach’s α if the item were to be deleted.

## Results

### Phase 2

#### The samples

A total of 2835 participants completed the survey. A larger proportion of participants completed the survey online (69%). Table 2 details the participant characteristics. T1DM participants were younger than T2DM (as expected). At time point 1, there was a higher proportion of female T1DM than male T1DM respondents, whereas there were similar numbers of male and female T2DM respondents. The HASMID^v1^ scores were derived from the original eight HASMID^v1^ items. The HASMID^v1^ questionnaire demonstrated a difference in mean utility scores from T1DM and T2DM respondents. T1DM reported lower QoL compared to T2DM.

**Table 2:** Participant characteristics

	Time point 1 (%)		Time point 2 (%)	
	T1DM (*n* = 795)	T2DM (*n* = 1989)	T1DM (*n* = 264)	T2DM (*n* = 959)
**Gender**				
Male	31.61	51.46	37.5	59.02
Female	68.39	48.39	62.50	40.88
Transgender	0.0	0.15	0	0.10
**Age**				
Mean (SD)	42.68 (15.97)	61.97 (11.64)	47.82 (15.83)	65.91 (11.15)
**Duration of DM**				
Mean (SD)	19.99 (14.29)	8.80 (6.81)	23.62 (16.02)	9.82 (7.00)
**Marital status**				
Single	26.54	10.16	21.21	8.86
Married/partner	64.03	71.04	69.70	70.07
Separated/divorced	5.41	10.21	4.92	9.80
Widowed	2.52	8.04	3.79	10.95
Other	1.51	0.55	0.38	0.31
**Education**				
Degree or equivalent professional qualification	49.69	36.75	54.92	40.25
**Employment**				
Employment/self-employment	62.52	33.43	58.71	25.65
**QoL**				
HASMID^v1^ utility score (SD)	0.57 (0.19)	0.70 (0.19)	0.62 (0.19)	0.75 (0.17)
EQ-5D-5L score (SD)	0.79 (0.22)	0.76 (0.25)	0.82 (0.20)	0.79 (0.23)

HASMID^v1^ utility score generated using Rowen et al., 2018 [5]; EQ-5D-5L generated using Devlin et al., 2016 [14]

The summary of questionnaire items are shown in Table 3, including floor and ceiling effects.

**Table 3:** Summary of questionnaire items

	T1DM (*n* = 795)				T2DM (*n* = 1989)				Difference in means between groups	Effect size using Cohen’s d	Wilcoxon rank-sum test (*p* value)	Statistically significant difference between groups (*p* < 0.05)
	Mean Item Score	(SD)	Floor (%)	Ceiling (%)	Mean Item Score	(SD)	Floor (%)	Ceiling (%)				
Temper	2.22	0.75	8.05	11.19	1.99	0.74	5.58	21.92	0.22	0.30	< 0.001	< 0.001
Irritable	2.23	0.59	4.65	3.90	2.08	0.57	3.47	9.30	0.15	0.25	< 0.001	< 0.001
Hypo	2.26	0.92	13.22	19.27	1.47	0.71	2.72	62.61	0.79	1.03	< 0.001	< 0.001
Tired	2.87	0.84	27.80	1.38	2.68	0.84	21.64	2.67	0.19	0.22	< 0.001	< 0.001
Tied meal times	2.01	0.96	8.05	37.86	1.86	0.92	4.84	45.39	0.15	0.16	< 0.001	< 0.001
Affects meal times	2.11	0.87	8.93	23.40	1.69	0.82	4.13	49.65	0.42	0.50	< 0.001	< 0.001
Control	1.98	0.86	6.67	31.45	1.79	0.81	4.38	40.76	0.19	0.23	< 0.001	< 0.001
Daily routine	2.34	0.90	14.47	14.72	1.67	0.79	4.08	48.41	0.66	0.81	< 0.001	< 0.001
Social activities	1.83	0.80	4.28	36.98	1.52	0.74	3.17	59.49	0.31	0.41	< 0.001	< 0.001
Hassle	2.59	0.86	18.24	7.04	1.84	0.79	4.03	36.44	0.75	0.92	< 0.001	< 0.001
Problem	2.23	0.73	8.30	9.18	1.77	0.70	3.17	35.90	0.47	0.66	< 0.001	< 0.001
Stressful	2.48	0.87	14.72	10.31	1.84	0.78	4.03	35.58	0.64	0.79	< 0.001	< 0.001
Support	2.40	0.81	6.67	14.09	2.16	0.89	6.25	26.68	0.24	0.28	< 0.001	< 0.001

Response items were coded never = 1; sometimes = 2; usually = 3; and always = 4. A greater item score indicates a greater deterioration in QoL

#### Validity

Validity of the HASMID items (both the original and alternatively phrased items) was assessed by examining known-group differences for each item across T1DM and T2DM respondents reported for time point 1. All items demonstrated statistically significant differences between T1DM and T2DM respondents. Significiant effect sizes were noted for Temper, Hypo, Daily Routine, Affects Mealtimes, Daily Routine, Social Activities, Hassle, Problem and Stressful.

### Summary of Rasch analysis

Full results of the DIF analyses can be found in Table 4. Full results of the DIF analyses are reported in Additional Material, Tables 3, 4, 5 and 6.

**Table 4:** Summary of Rasch analysis for random sample

	DIF gender	DIF age	DIF DM Type	Disordered Thresholds
Temper	✓	✓	✓	✘
Irritable	✓	✘	✘	✘
Hypo	✘	✘	✓	✓
Tired	✘	✘	✘	✘
Tied To Mealtimes	✘	✓	✓	✘
Affects Mealtimes	✓	✘	✘	✘
Control	✘	✘	✘	✘
Daily Routine	✘	✓	✓	✓
Social Activities	✘	✘	✘	✓
Hassle	✘	✓	✓	✘
Problem	✘	✘	✓	✘
Stressful	✘	✓	✓	✘
Support	✘	✘	✓	✘

✓ denotes present ✘ denotes absent

#### Comparing temper and irritable

Temper demonstrated DIF for gender, age and DM type. Irritable demonstrated DIF for gender. Neither item showed disordered thresholds.

#### Comparing hassle and problem

Hassle demonstrated DIF for age and DM type. Problem demonstrated DIF for DM type. Neither item showed disordered thresholds.

#### Comparing tied to mealtimes, affects mealtimes, daily routine and social activities

Tied To Mealtimes showed DIF for age and DM type Affects Mealtimes showed DIF for gender. Daily Routine showed DIF for age and DM type and disordered thresholds. Whilst Social Activities did not demonstrate any DIF, disordered thresholds were noted.

#### Examining hypo, tired, control, stressful and support

Hypo showed disordered thresholds and DIF for DM type. Tired and Control demonstrated no DIF or showed any disordered thresholds. Stressful demonstrated DIF for age and DM type. Support demonstrated DIF for DM type.

### Final item selection

Item selection was informed by the analyses reported above. Firstly, consideration was made on the alternative items. Finally, the remaining original HASMID items were discussed to determine whether there was sufficient evidence to remove any items from the questionnaire.

#### Temper versus irritable

Conceptually both items can be considered as “mood” items, with temper tapping into the more severe end of the spectrum. Having items that can measure at the extremes can be useful. The psychometric results showed both items were moderately correlated (results not reported). Temper was able to discriminate between the two respondent groups, as shown by a significant effect size. Both items also demonstrated DIF for gender, and Temper also demonstrated DIF for age and DM type. Clinical opinion was also considered, and whilst it was acknowledged that respondents are more likely to be aware of if they have experienced Temper (rather than irritability), temper itself could be considered as a personality trait. Similarly, there are possible social connotations of admitting to having experienced temper. Irritable is a milder item, that is unlikely to be a personality trait and there are no social connotations of feeling/being irritable. A decision was made to retain Temper as an item, and to include Irritable as an additional item.

#### Hassle versus problem

Both items appeared to perform similarly psychometrically, with similar levels of floor/ceiling effects and DIF. Given that there is no overwhelming evidence to support the notion to change the item from Hassle to Problem, the original wording was kept.

#### Tied to mealtimes versus affects mealtimes, daily routine, social activities

All four items appeared to have similar psychometric properties. Out of the four items, Social Activities item was preferred as it demonstrated no DIF compared to the other three items. From a clinical perspective there were concerns around the rigidity of concepts such as mealtimes and routine. It was felt that whilst these may be an issue to some, these could be generational concepts where older people may be more likely to follow a routine and have stricter mealtimes (as shown in the DIF analysis). In the longer term, these items could be redundant – people with DM now may not have a mealtime/routine in the way that someone of an older generation has. However, it was noted to be an important factor for PwD in QoL. A decision was made to include Social Activities as an additional item, as the Rasch analysis suggested this to be a good item and to address that DM and self-management are likely to impact upon more than just mealtimes per se.

#### Hypo, tired, control, stress, and support

The Hypo item is more relevant to people with T1DM as those with T2DM. Therefore, not unsurprisingly the floor and ceiling effects in psychometric analyses were better for those with T1DM as opposed to T2DM. This is reflected in the Rasch analysis as it demonstrated DIF for DM type. It’s inclusion in the final questionnaire is driven by the importance of the concept itself. The fear of “going hypo” will be more prevalent in those with T1DM, as virtually all people living with T1DM will have experienced hypoglycaemia. They will have to regularly self-adjust their medication to minimise the chances of hypoglycaemia, whilst also avoiding hyperglycaemia, in order to control their HbA1c levels and avoid long-term complications of diabetes. The same cannot be said for the majority of people living with T2DM. As whilst some people living with T2DM will have to adjust their medication, under direction of their GP/physician, to control their HbA1c levels; the majority will either follow a controlled diet alone, or a controlled diet with a tablet medication that does not put them at risk of hypoglycaemia. Only those people with more complex T2DM will need medication that can cause hypoglycaemia (if given inappropriately) [15]. Thus, the level of self-management engagement required for glucose control, to avoid hypo- and hyper-glycaemia, in people with T2DM is lower than T1DM. A decision was made to retain all items given insufficient evidence to suggest their removal. All concepts were felt to be clinically important.

### HASMID-10

The final measure, HASMID-10 (Table 5), consists of ten items that cover Temper, Irritable, Hypo, Tired, Tied To Mealtimes, Social Activities, Control, Hassle, Stress, and Support (the original HASMID^v1^ items plus Irritable and Social Activities). The response options for the HASMID-10 are that of the original PRO (never, sometimes, usually, and always). The overall questionnaire is reverse scored summatively, with response levels being scored as never = 3, sometimes = 2, usually = 1, always = 0. Scores can range from 0 to 30, with a higher score indicating better quality of life. Utility scores and willingness-to-pay values can be generated for HASMID-10, for each time a respondent completes the measure [5, 6]. The utility scores generate a utility value each time a respondent completes the measure, and can be used to generate QALYs for cost-utility analyses for economic evaluation to inform resource allocation decisions. The willingness-to-pay values generate a monetary value each time a respondent completes the measure, and can be used to generate a monetary value of the benefit of treatments for use in cost-benefit analyses for economic evaluation.

**Table 5:** HASMID-10 questionnaire

Mood	You never find yourself losing your temper over small things
	You sometimes find yourself losing your temper over small things
	You usually find yourself losing your temper over small things
	You always find yourself losing your temper over small things
Mood	You are never irritable
	You are sometimes irritable
	You are usually irritable
	You are always irritable
Hypoglycaemic attacks	You never worry about going hypo
	You sometimes worry about going hypo
	You usually worry about going hypo
	You always worry about going hypo
Vitality	You are never tired
	You are sometimes tired
	You are usually tired
	You are always tired
Social Limitations	Your days are never tied to meal times
	Your days are sometimes tied to meal times
	Your days are usually tied to meal times
	Your days are always tied to meal times
Social Limitations	Your diabetes never limits your social activities
	Your diabetes sometimes limits your social activities
	Your diabetes usually limits your social activities
	Your diabetes always limits your social activities
Control	You feel you have a lot of control of your diabetes
	You feel you have some control of your diabetes
	You feel you have little control of your diabetes
	You feel you have no control of your diabetes
Hassle	You find your life with diabetes is never a hassle
	You find your life with diabetes is sometimes a hassle
	You find your life with diabetes is often a hassle
	You find your life with diabetes is always a hassle
Stress	You find your life with diabetes is never stressful
	You find your life with diabetes is sometimes stressful
	You find your life with diabetes is often stressful
	You find your life with diabetes is always stressful
Support	You feel totally supported with your diabetes
	You feel you have a lot of support with your diabetes
	You feel you have a little support with your diabetes
	You feel you have no support with your diabetes

#### Internal consistency

The internal consistency of the questionnaire for the entire questionnaire was 0.84. Table 6 details inter-item covariance and Cronbach’s α scores if the item were to be deleted.

**Table 6:** Internal consistency results for HASMID-10

Item	Average item covariance	Cronbach’s alpha if item deleted
Temper	0.25	0.83
Irritable	0.25	0.83
Hypo	0.24	0.83
Tired	0.24	0.83
Tied To Mealtimes	0.26	0.86
Control	0.23	0.83
Social Activities	0.23	0.82
Hassle	0.21	0.81
Stressful	0.21	0.81
Support	0.24	0.84

## Discussion

In the development of any PRO it is important to fully evaluate its performance prior to mainstream usage. The present study has allowed us to further improve on the face and content validity of the HASMID instrument. The original questionnaire was developed using a mixed methods approach, with items generated from interviews with PwD and an existing PRO measure [4]. Here we have been able to re-examine the content of the HASMID questionnaire through Patient and Public Involvement consultation and two focus group cognitive debriefing exercises. The alternatively phrased items were then subjected to psychometric evaluation alongside the existing HASMID items.

A limitation of the study is the method of data collection. Every effort was made to ensure that participants did not complete the online survey twice (to increase their chances in the prize draw). We removed responses within the same time period that provided a duplicate email address. However, it was not possible to identify people who may have completed the survey twice in the same period who used different email addresses. We did note that the sociodemographic and health profiles of PwD differed depending upon whether they completed the online or postal survey. The implications of this are discussed further in Rowen et al. (2019) [16]. Participants were self-selecting, and this too could be considered a limitation.

The development, refinement and evaluation of PRO instruments can be driven by different theoretical approaches: Classical Test Theory (CTT) Item Response Theory (IRT) and Rasch Measurement Theory (RMT) [17]. Here we applied CTT and RMT to assess the performance of the alternatively worded items. Both assessments were used, in conjunction with clinical opinion to inform the final selection of items. This approach has been used by others in PRO development [18, 19]. There are benefits to considering alternative sources of evidence. One of the main indicators for the inclusion of Rasch analysis in this study was to identify whether items demonstrated DIF. There is logic to considering eliminating items based upon differing responses driven by gender and age. However, in the context of self-management of diabetes, age and gender may well be factors that drive individuals’ responses. For example, being tied to mealtimes may be a negative issue for the younger generation, whereas older people may already follow a more structured routine. The benefit of including psychometric analyses, Rasch analyses and clinical opinion allows a consideration of the relevance of the inclusion/exclusion of each item. Adopting only one approach may result in an instrument that is not relevant to the target population or provide information that is not useful in trials, service evaluations, or routine clinical care.

The alternative items to the original HASMID items tested in this study were felt to be exploring the same concept, but with slightly different phrasing. For three (of the five) alternatively phrased items there were insufficient evidence to suggest an amendment to the wording of the original HASMID item. Two of the alternative items have now been incorporated into the HASMID-10 questionnaire – Irritable and Social Activities. Both items performed well psychometrically, were deemed of clinical importance and captured different severity of underlying health to the original items.

One of the limitations of the study was that respondents self-reported information about their diabetes including diabetes type, duration of diabetes, HbA1c status, treatment and diabetes-related complications. Our HbA1c data indicates that respondents often did not know their HbA1c status, meaning that this cannot be reliably used to assess how HASMID performs across different levels of HbA1c, and we have no objective measure of severity by which to examine the items.

A potential limitation to the study is the applicability and performance of items across different ethnic groups. Within the psychometric survey information on ethnicity was not collected. This was a purposeful omission. It was outside the scope of the study to assess the cultural validity of the overall questionnaire and all potential items. To do so would require further qualitative work across different ethnic groups, specifically on cognitive debriefing to ensure items are relevant, and to identify any potential new items for consideration. Therefore, it was felt that to collect data on the ethnicity of survey respondents would not be relevant. Any difference in HASMID scores across groups could not be validated at this stage. Further research is required to assess the cross-cultural validity of the HASMID-10 amongst different populations.

## Conclusion

This cross-sectional validation study has examined the psychometric properties of the original HASMID items and tested potential items for inclusion using a large dataset. Rasch analysis was undertaken and considered alongside conventional psychometric performance and clinical opinion. The analyses found the items to have good psychometric performance, with discriminative validity to be able to discriminate across type of diabetes. However further assessment of psychometric performance is recommended by administering the measure alongside a clinical intervention. The final HASMID questionnaire now consists of ten items, the HASMID-10. The additional two items may provide further insight into how PwD are self-managing their condition, by providing further detail into how emotions and daily activities are affected.

## Supplementary information

**Additional file 1: Table 1.** Focus group participants. **Box 1.** Summary of suggestions made by focus group participants on the layout and instructions for the HASMIDv1 questionnaire. **Table 2.** Summary of comments on HASMIDv1 questionnaire items. **Table 3.** DIF analysis results comparing Temper (original HASMIDv1 item) and Irritable (alternative item). **Table 4.** DIF analysis results comparing Hassle (original HASMIDv1 item) and Problem (alternative item). **Table 5.** DIF analysis results Comparing Tied Mealtimes (original HASMIDv1 item), Affects Mealtimes (alternative item), Daily Routine (alternative item) and Social Activities (alternative item). **Table 6.** DIF analysis Comparing Hypo, Tired, Control, Stressful, Support.

## Data Availability

The datasets during and/or analysed during the current study available from the corresponding author on reasonable request.
