# Supplementary material for: Assessment of the psychometric properties and refinement of the Health and Self-Management in Diabetes Questionnaire (HASMID)
Source: Health Qual Life Outcomes. 2020 Mar 5;18:59. doi: 10.1186/s12955-020-01305-3 (PMC7059394; doi:10.1186/s12955-020-01305-3)
Supplement: Supplementary file 1 — Additional file 1: Table 1. Focus group participants. Box 1. Summary of suggestions made by focus group participants on the layout and instructions for the HASMIDv1 questionnaire. Table 2. Summary of comments on HASMIDv1 questionnaire items. Table 3. DIF analysis results comparing Temper (original HASMIDv1 item) and Irritable (alternative item). Table 4. DIF analysis results comparing Hassle (original HASMIDv1 item) and Problem (alternative item). Table 5. DIF analysis results Comparing Tied Mealtimes (original HASMIDv1 item), Affects Mealtimes (alternative item), Daily Routine (alternative item) and Social Activities (alternative item). Table 6. DIF analysis Comparing Hypo, Tired, Control, Stressful, Support. [file 12955_2020_1305_MOESM1_ESM.docx]

**Additional Material**

Table 1 Focus group participants

|  | **Gender** | | **Age (years)** | | | | **Diabetes Type** | |
| --- | --- | --- | --- | --- | --- | --- | --- | --- |
|  | **Male** | **Female** | **50-59** | **60-69** | **70-79** | **80-89** | **T1DM** | **T2DM** |
| Focus Group 1 (n=7) | 4 | 3 | 2 | 0 | 4 | 1 | 2 | 5 |
| Focus Group 2 (n=8) | 6 | 2 | 1 | 3 | 3 | 1 | 4 | 4 |

T1DM = Type 1 Diabetes Mellitus T2DM = Type 2 Diabetes Mellitus

Box 1 Summary of suggestions made by focus group participants on the layout and instructions for the HASMID^v1^ questionnaire

| - Felt the instructions for the questionnaire need personalising - Liked the spacing between the lines (between response options) - Felt there needs to be more emphasis on the effect diabetes has had, rather than other long-term conditions - Some discussion on whether recall period should be increased to one month - Wanted larger font |
| --- |

Table 2 Summary of comments on HASMID^v1^ questionnaire items

| **Original HASMID^v1^ questionnaire item** | **Comments** | **Suggested changes** |
| --- | --- | --- |
| Mood | - Felt *losing temper* was too extreme - Felt it was an appropriate question to ask   Discussed whether it was too severe | - Suggested *irritable* |
| Hypoglycaemic attacks | - Felt *worry* was too extreme - Some stated that some people would not know what a hypoglycaemic (hypo) attack was - Some asked why not have question about a hyperglycaemic attack - Long discussion about what if you don’t know what a hypo is   Some felt the question was more appropriate to T1DM than T2DM, others disagreed | - Suggested *concerned* - Suggested *anxious* - Suggested having a description of what a hypo is |
| Vitality | Nil | Suggested *energy levels* as a title |
| Social limitations | - Didn’t like the title *social limitations* - Noted that some people lead a very regimented lifestyle and like to have fixed mealtimes (even before they were diagnosed with diabetes) | - Suggestions of *eating habits* or *daily routine* or *day-to-day living* - Liked *does your diabetes ever interfere with your meal times* |
| Control | Nil | Nil |
| Hassle | - Some concerns that people wouldn’t know what hassle meant - Felt the word hassle was not appropriate | - Suggested “problem” or “problems” - Suggested problem as an alternative |
| Stress | Nil | Nil |
| Support (All support you have, including Clinical and Personal) | - Wondered if needed two separate questions to cover clinical and personal |  |

Table 3 DIF analysis results comparing Temper (original HASMID^v1^ item) and Irritable (alternative item)

|  | **Class interval** | | | | | | | **Gender** | | | | | | | | | | **Class interval by gender** | | | | | | | | |
| --- | --- | --- | --- | --- | --- | --- | --- | --- | --- | --- | --- | --- | --- | --- | --- | --- | --- | --- | --- | --- | --- | --- | --- | --- | --- | --- |
|  | MS | F | | DF | | Prob | | MS | | F | | DF | | | Prob | | | MS | | F | DF | | | Prob | | |
| Temper | 310.95 | 736.17 | | 2 | | 0.00 | | 4.81 | | 6.37 | | 1 | | | 0.01 | | | 4.50 | | 5.97 | 2 | | | 0.00 | | |
| Irritable | 41.76 | 11.41 | | 2 | | 0.00 | | 13.69 | | 3.74 | | 1 | | | 0.05 | | | 30.04 | | 8.21 | 2 | | | 0.00 | | |
|  | **Class interval** | | | | | | | **Age** | | | | | | | | | | **Class interval by age** | | | | | | | | |
|  | MS | F | | DF | | Prob | | MS | F | | | | DF | | | Prob | | MS | F | | | DF | | | Prob | |
| Temper | 332.07 | 712.27 | | 2 | | 0.00 | | 31.41 | 40.38 | | | | 4 | | | 0.00 | | -16.58 | -21.31 | | | 7 | | | 1.00 | |
| Irritable | 40.84 | 10.72 | | 2 | | 0.00 | | 3.35 | 0.88 | | | | 4 | | | 0.48 | | 3.28 | 0.86 | | | 7 | | | 0.54 | |
|  | **Class interval** | | | | | | | **DM Type** | | | | | | | | | | **Class interval by DM Type** | | | | | | | | |
|  | MS | | F | | DF | | Prob | MS | | | F | | | DF | | | Prob | MS | | F | | | DF | | | Prob |
| Temper | 1310.5 | | 1660.0 | | 2 | | 0.00 | 19.79 | | | 25.07 | | | 1 | | | 0.00 | -9.25 | | -11.72 | | | 2 | | | 1.00 |
| Irritable | 40.10 | | 10.36 | | 2 | | 0.00 | 1.35 | | | 0.35 | | | 1 | | | 0.55 | 0.70 | | 0.18 | | | 2 | | | 0.83 |

Table 4 DIF analysis results comparing Hassle (original HASMID^v1^ item) and Problem (alternative item)

|  | **Class interval** | | | | | | | **Gender** | | | | | | | **Class interval by gender** | | | | | |
| --- | --- | --- | --- | --- | --- | --- | --- | --- | --- | --- | --- | --- | --- | --- | --- | --- | --- | --- | --- | --- |
|  | MS | | F | DF | | Prob | | MS | | F | DF | | Prob | | MS | | F | | DF | Prob |
| Hassle | 2.20 | | 6.40 | 2 | | 0.00 | | 1.21 | | 3.50 | 1 | | 0.06 | | 0.50 | | 1.45 | | 2 | 0.24 |
| Problem | 2.69 | | 6.37 | 2 | | 0.00 | | 1.66 | | 3.93 | 1 | | 0.05 | | 0.55 | | 1.30 | | 2 | 0.27 |
|  | **Class interval** | | | | | | | **Age** | | | | | | | **Class interval by age** | | | | | |
|  | MS | | F | DF | | Prob | | MS | | F | DF | | Prob | | MS | | F | | DF | Prob |
| Hassle | 2.20 | | 6.62 | 2 | | 0.00 | | 1.19 | | 3.57 | 4 | | 0.01 | | 0.58 | | 1.75 | | 8 | 0.09 |
| Problem | 2.69 | | 6.55 | 2 | | 0.00 | | 1.30 | | 3.15 | 4 | | 0.01 | | 0.69 | | 1.68 | | 8 | 0.10 |
|  | **Class interval** | | | | | | | **DM Type** | | | | | | | | **Class interval by DM Type** | | | | |
|  | MS | F | | | DF | | Prob | MS | F | | | DF | | Prob | | MS | | F | DF | Prob |
| Hassle | 2.22 | 6.79 | | | 2 | | 0.00 | 4.90 | 15.0 | | | 1 | | 0.00 | | 1.37 | | 4.19 | 2 | 0.02 |
| Problem | 2.65 | 6.48 | | | 2 | | 0.00 | 4.97 | 12.2 | | | 1 | | 0.00 | | 0.95 | | 2.33 | 2 | 0.10 |

Table 5 DIF analysis results Comparing Tied Mealtimes (original HASMID^v1^ item), Affects Mealtimes (alternative item), Daily Routine (alternative item) and Social Activities (alternative item)

|  | **Class interval** | | | | | | | | | **Gender** | | | | | | | | | | **Class interval by gender** | | | | |
| --- | --- | --- | --- | --- | --- | --- | --- | --- | --- | --- | --- | --- | --- | --- | --- | --- | --- | --- | --- | --- | --- | --- | --- | --- |
|  | MS | F | | DF | | | Prob | | | MS | | F | | | DF | | | Prob | | MS | F | | DF | Prob |
| Tied Mealtimes | 8.22 | 7.93 | | 2 | | | 0.00 | | | 1.01 | | 0.97 | | | 1 | | | 0.32 | | -0.11 | -0.11 | | 2 | 1.00 |
| Affects Mealtimes | 4.76 | 7.44 | | 2 | | | 0.00 | | | 7.03 | | 10.99 | | | 1 | | | 0.00 | | 0.10 | 0.15 | | 2 | 0.86 |
| Daily Routine | 3.95 | 6.40 | | 2 | | | 0.00 | | | 0.37 | | 0.59 | | | 1 | | | 0.44 | | 0.08 | 0.13 | | 2 | 0.87 |
| Social Activities | 4.50 | 7.47 | | 2 | | | 0.00 | | | 3.88 | | 6.43 | | | 1 | | | 0.01 | | 0.61 | 1.00 | | 2 | 0.37 |
|  | **Class interval** | | | | | | | | | **Age** | | | | | | | | | | **Class interval by age** | | | | |
|  | MS | | F | | | DF | | | Prob | MS | | | F | | | DF | | | Prob | MS | | F | DF | Prob |
| Tied Mealtimes | 8.74 | | 9.70 | | | 2 | | | 0.00 | 17.20 | | | 19.10 | | | 4 | | | 0.00 | -0.67 | | -0.75 | 8 | 1.00 |
| Affects Mealtimes | 4.93 | | 7.56 | | | 2 | | | 0.00 | 0.85 | | | 1.31 | | | 4 | | | 0.27 | 0.61 | | 0.93 | 8 | 0.49 |
| Daily Routine | 4.11 | | 7.85 | | | 2 | | | 0.00 | 10.78 | | | 20.60 | | | 4 | | | 0.00 | -0.18 | | -0.34 | 8 | 1.00 |
| Social Activities | 4.60 | | 7.56 | | | 2 | | | 0.00 | 1.50 | | | 2.47 | | | 4 | | | 0.04 | 2.44 | | 0.40 | 8 | 0.92 |
|  | **Class interval** | | | | | | | | | **DM Type** | | | | | | | | | | **Class interval by DM Type** | | | | |
|  | MS | F | | | DF | | | Prob | | MS | F | | | DF | | | Prob | | | MS | F | | DF | Prob |
| Tied Mealtimes | 8.36 | 8.35 | | | 2 | | | 0.00 | | 15.04 | 15.02 | | | 1 | | | 0.00 | | | 1.20 | 1.20 | | 2 | 0.30 |
| Affects Mealtimes | 4.50 | 7.08 | | | 2 | | | 0.00 | | 0.01 | 0.01 | | | 1 | | | 0.92 | | | 0.23 | 0.37 | | 2 | 0.69 |
| Daily Routine | 3.97 | 7.22 | | | 2 | | | 0.00 | | 25.93 | 47.13 | | | 1 | | | 0.00 | | | 0.28 | 0.51 | | 2 | 0.60 |
| Social Activities | 4.90 | 8.09 | | | 2 | | | 0.00 | | 0.01 | 0.01 | | | 1 | | | 0.93 | | | 0.79 | 1.31 | | 2 | 0.27 |

Table 6 DIF analysis Comparing Hypo, Tired, Control, Stressful, Support

|  | **Class interval** | | | | | | | **Gender** | | | | | | | | | **Class interval by gender** | | | | | | |
| --- | --- | --- | --- | --- | --- | --- | --- | --- | --- | --- | --- | --- | --- | --- | --- | --- | --- | --- | --- | --- | --- | --- | --- |
|  | MS | | F | | DF | | Prob | MS | | F | | DF | | | Prob | | MS | F | | DF | | Prob | |
| Hypo | 0.12 | | 0.15 | | 2 | | 0.86 | 2.17 | | 2.78 | | 1 | | | 0.10 | | 0.34 | 0.43 | | 2 | | 0.65 | |
| Tired | 0.21 | | 0.28 | | 2 | | 0.76 | 2.29 | | 2.95 | | 1 | | | 0.09 | | 0.01 | 0.01 | | 2 | | 0.99 | |
| Control | 4.66 | | 7.00 | | 2 | | 0.00 | 0.15 | | 0.22 | | 1 | | | 0.64 | | 1.19 | 1.80 | | 2 | | 0.17 | |
| Stressful | 12.18 | | 22.96 | | 2 | | 0.00 | 0.10 | | 0.19 | | 1 | | | 0.66 | | 0.86 | 1.62 | | 2 | | 0.20 | |
| Support | 8.83 | | 9.73 | | 2 | | 0.00 | 1.88 | | 2.08 | | 1 | | | 0.15 | | 1.23 | 1.38 | | 2 | | 0.25 | |
|  | **Class interval** | | | | | | | **Age Group** | | | | | | | | | **Class interval by age group** | | | | | | |
|  | MS | | F | | DF | | Prob | MS | | F | DF | | | Prob | | | MS | F | | DF | | Prob | |
| Hypo | 0.11 | | 0.15 | | 2 | | 0.86 | 1.93 | | 2.48 | 4 | | | 0.04 | | | 0.27 | 0.35 | | 8 | | 0.95 | |
| Tired | 0.22 | | 0.28 | | 2 | | 0.76 | 1.05 | | 1.37 | 4 | | | 0.25 | | | 1.05 | 1.37 | | 8 | | 0.21 | |
| Control | 4.52 | | 6.83 | | 2 | | 0.00 | 0.59 | | 0.89 | 4 | | | 0.47 | | | 1.03 | 1.55 | | 8 | | 0.14 | |
| Stressful | 12.32 | | 23.50 | | 2 | | 0.00 | 4.84 | | 9.24 | 4 | | | 0.00 | | | -1.21 | -2.31 | | 8 | | 1.00 | |
| Support | 8.83 | | 9.63 | | 2 | | 0.00 | 1.34 | | 1.46 | 4 | | | 0.21 | | | 0.32 | 0.35 | | 8 | | 0.95 | |
|  | **Class interval** | | | | | | | **DM Type** | | | | | | | | | **Class interval by DM Type** | | | | | | |
|  | MS | F | | DF | | Prob | | MS | F | | | | DF | | | Prob | MS | | F | | DF | | Prob |
| Hypo | 0.28 | 0.42 | | 2 | | 0.66 | | 42.89 | 64.53 | | | | 1 | | | 0.00 | 2.24 | | 3.37 | | 2 | | 0.04 |
| Tired | 0.27 | 0.36 | | 2 | | 0.70 | | 3.81 | 5.10 | | | | 1 | | | 0.02 | 0.86 | | 1.15 | | 2 | | 0.32 |
| Control | 4.75 | 7.25 | | 2 | | 0.00 | | 4.69 | 7.17 | | | | 1 | | | 0.01 | 1.92 | | 2.93 | | 2 | | 0.05 |
| Stressful | 11.96 | 23.03 | | 0 | | 0.00 | | 7.42 | 14.30 | | | | 1 | | | 0.00 | -1.45 | | -2.80 | | 2 | | 1.00 |
| Support | 7.98 | 8.89 | | 2 | | 0.00 | | 12.05 | 13.42 | | | | 1 | | | 0.00 | -0.94 | | -1.05 | | 2 | | 1.00 |
